# Quality of life in older patients with cancer and related unmet needs: a scoping review

**DOI:** 10.2340/1651-226X.2025.42602

**Published:** 2025-04-15

**Authors:** Franziska Springer, Ayumu Matsuoka, Kyoko Obama, Anja Mehnert-Theuerkauf, Yosuke Uchitomi, Maiko Fujimori

**Affiliations:** a Department of Medical Psychology and Medical Sociology, Comprehensive Cancer Center Central Germany (CCCG), University Medical Center Leipzig, Leipzig, Germany; b Division of Survivorship Research, National Cancer Center, Institute for Cancer Control, Tokyo, Japan; c Department of Cancer Survivorship and Digital Medicine, The Jikei University School of Medicine, Tokyo, Japan

**Keywords:** cancer, quality of life, older adults, unmet needs, geriatric

## Abstract

**Background:**

Older patients form the largest group of cancer patients yet remain underrepresented in clinical research. This scoping review aims to synthesize findings on quality of life (QoL) in older adults with cancer, comparing them to younger counterparts and older individuals without cancer, and identifying associated factors.

**Methods:**

PubMed and PsychINFO databases were searched for articles published until January 2024. Studies were included with exclusively older adults with cancer (≥ 65 years), age-mixed samples (mean/median ≥ 70 years), or that report results separately for older and younger adults with cancer. Out of 6, 397 identified studies, 87 met the inclusion criteria.

**Results:**

Most studies were cross-sectional, conducted in 14 countries with a mean age of 74.2 years. Physical QoL (PQoL) demonstrates an age-related decline, primarily influenced by comorbidity burden, physical activity, and lifestyle. In contrast, mental QoL (MQoL) remained stable or increased with advancing age, reflecting resilience and effective coping by older patients. While cognitive and role functioning tended to show stable or declining values with age, findings regarding social functioning were mixed. Socioeconomic factors, e.g. education, income, or marital status, mainly impacted MQoL, as well as other QoL domains. Symptom management and social support represent unmet needs that contribute to QoL impairments. Older adults with cancer underreport symptoms they perceive as normal for their age, experience ageism in healthcare, and reduced social participation.

**Interpretation:**

Comprehensive, multidisciplinary cancer care is essential for older adults with cancer, focusing on the prevention of functional health decline, geriatric assessment, socioeconomic health disparities, and enhancing symptom management.

## Introduction

The impact of a cancer diagnosis on patients’ Quality of Life (QoL) has been extensively studied [1–3]. QoL is understood to encompass physical, emotional, social, role and cognitive aspects [4]. Impairments in QoL dimensions, such as reduced social functioning (SF) due to a fear of stigmatization, social isolation, or changes in intimate relationships, vary considerably between patients and are dependent on the individual situation.

Despite the numerous studies on QoL within the oncological context, results on older adults with cancer are very limited. Older patients already represent the largest proportion of cancer patients, with more than two thirds of newly diagnosed cancer patients being 60 years or older [5, 6]. The supportive care needs and QoL of older patients may differ substantially from those of younger patients, yet older adults with cancer are highly underrepresented in clinical research [7, 8]. In addition, the assessment of QoL in older adults is impeded since well-known QoL assessment tools have been developed and validated for adult cancer patients of all ages and may lack important geriatric aspects specific to older individuals [9, 10].

Managing a cancer disease in older patients is often complex due to high rates of physical comorbidity, potential polypharmacy, small social support networks, widowhood, financial constraints, cognitive decline, or impairments of frailty and mobility [11, 12]. This results in a heterogeneous cohort of patients with disparate health and geriatric concerns that must be considered when planning supportive care and targeting treatment. Treating clinicians, however, frequently base their treatment decisions on data obtained from younger and healthier patients. Expanding the evidence-based knowledge base for older adults with cancer could enhance supportive care.

Ongoing demographic changes worldwide, combined with better cancer treatments and prolonged survival, pose an increasing challenge to healthcare systems in supporting older cancer survivors with their QoL-related supportive care needs [13]. Older adults with cancer are often subdivided into the ‘young-old’ (65–74 years), ‘middle-old’ (75–84 years) and ‘old-old’ (≥ 85 years) [14] with distinct challenges and care needs. To enhance survivorship care planning and treatment, a robust evidence base on QoL in older adults with cancer is needed [15]. This will help to clarify their specific unmet needs and functional health impairments and to identify vulnerable subgroups at risk for reduced QoL. A preliminary search for the existing reviews on QoL in older adults with cancer revealed few results, which were either not up-to date, or targeted specific subpopulations [16–18]. A comprehensive review on QoL in all older adults with cancer is missing to date.

The aim of this scoping review therefore was to describe important dimensions of QoL, related unmet needs and functional health aspects in older adults with cancer in comparison to younger cancer patients and older non-cancer cases, and to identify medical, sociodemographic, psychosocial and geriatric factors associated with QoL.

## Methods

The PRISMA-ScR guidelines [19] were followed for this scoping review (Supplementary Table SI). The study was registered at Open Science Framework (osf.io/2vu9x), and the study protocol has been published [20].

### Inclusion and exclusion criteria

Study selection was based on PCC (population, concept, context) framework recommended by JBI [21] to identify relevant studies. The population of interest consists of older adults with cancer across all tumor entities, healthcare settings and treatment stages. We included studies that addressed exclusively older adults with cancer (≥ 65 years), or age-mixed samples with either mean/median age of ≥ 70 years, and thus mainly consist of older patients, or that report results separately for older and younger cancer cancer patients. The types of sources were limited to studies that report results on validated QoL assessment tools. We excluded reviews, meta-analyses, intervention studies with QoL as outcome, case studies, case series, opinion pieces, editorials, study protocols and conference articles.

The concepts of interest were dimensions of QoL in older adults with cancer including QoL-related unmet needs and functional health. We included studies with a comparison of QoL dimensions in older adults with cancer to older cancer-free individuals or population norms, and to younger cancer patients (<65 years). Lastly, studies that identified sociodemographic, medical, geriatric and psychological factors associated with QoL dimensions in older adults with cancer were included.

### Search strategies

**Figure 1:**
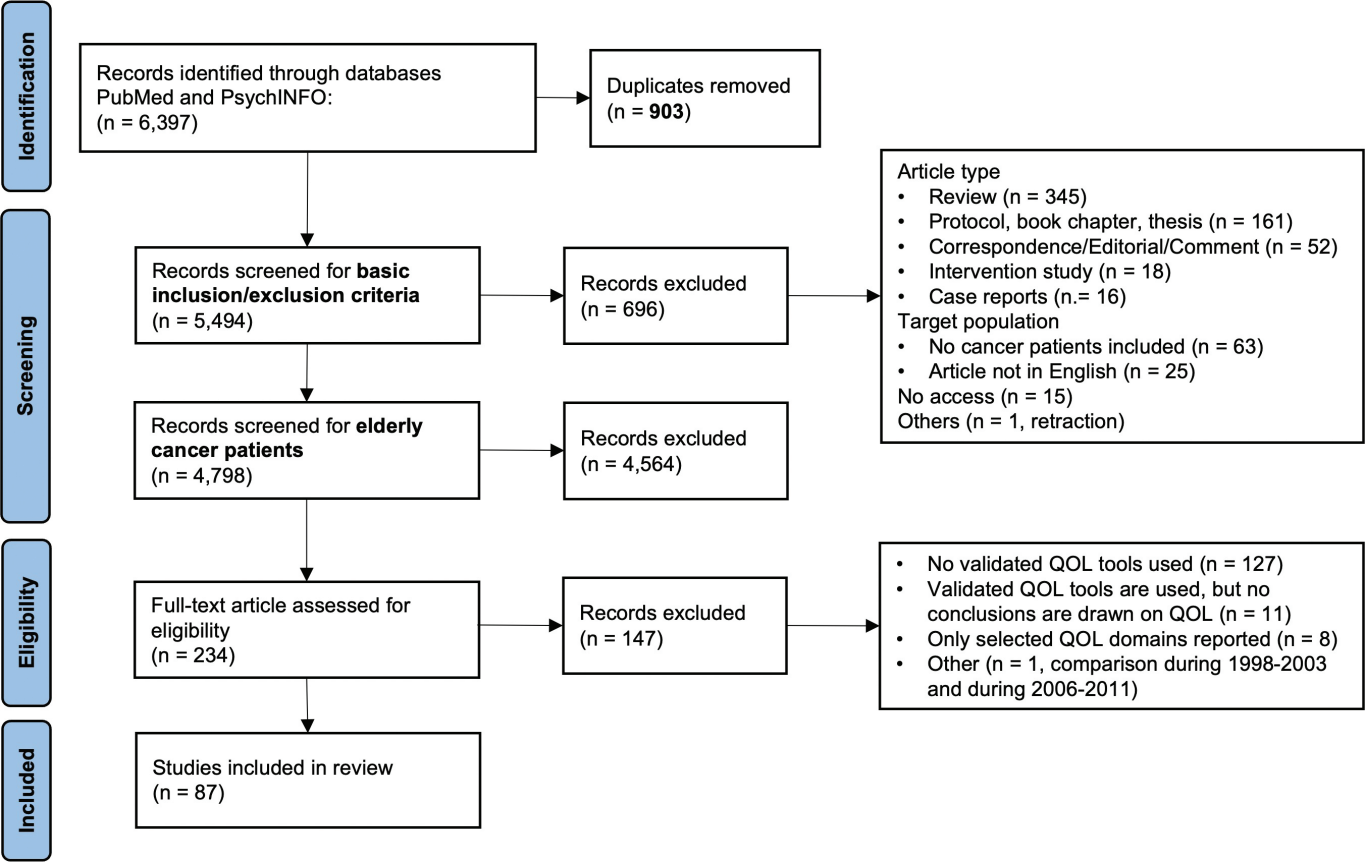
PRISMA flow-chart.

A comprehensive literature search of studies published in English language until January 2024 was conducted using PubMed and PsychINFO. The search strategy was built on relevant key words around cancer and QoL. For more details on search terms and study selection, see Table 1 and published study protocol [20]. The search identified 6, 397 studies, of which 903 duplicates were removed. After screening titles and abstracts for basic inclusion criteria, 4, 798 studies remained that were checked for our age criteria, of which 4, 564 were excluded that did not address older adults with cancer. The remaining 234 articles were full text reviewed by the first and second author (FS, AM), resulting in 145 exclusions, mainly because no validated QoL assessment tool was used. Any disagreements regarding the inclusion of studies were resolved through discussion within the team of authors. Finally, 87 articles were considered for this scoping review (Figure 1).

**Table 1:** A summary of scoping review procedure.

**Study population**	Cancer patients of all tumor entities were included, focusing exclusively on older adults with cancer (≥ 65 years), or, for age-mixed samples, with a mean/median age ≥ 70 years, 70 years, or that report results separatley for older and younger cancer patients
**Concept**	Studies reporting on (1) outcomes of validated QoL assessment tools (global QoL and dimensions), (2) comparing QoL dimensions of younger and older adults with cancer, or older adults with cancer and older non-cancer controls, and (3) identifying sociodemographic, medical, geriatric and psychological factors associated with QoL
**Context**	All countries, regions, and health care settings
**Types of sources**	
** Inclusion**	Published articles in English language presenting quantitative, qualitative or mixed methods studies
** Exclusion**	Reviews, meta-analyses, intervention studies with QoL as outcome, case studies, case series, opinion pieces, editorials, study protocols and conference papers
**Search terms**	(‘Survivors’[Major] OR ‘Survivors/psychology’[Major]) AND (‘neoplasms’[Major] OR ‘Carcinoma’[Major]) AND (‘Quality of Life’[Mesh] OR ‘patient-reported outcomes’ OR ‘health-related quality of life’ OR ‘wellbeing’ OR ‘well-being’ OR ‘Mental Health’[Major] OR ‘Physical Fitness/psychology’[Major] OR ‘Physical Fitness/Physiology’[Major] OR ‘Health Status’[Major] OR ‘late effects’) AND adults

QoL: quality of life.

### Data extraction

The data were extracted by FS using a custom data extraction sheet. For quality checking, 10% of the studies were checked by AM for accuracy. The following information was extracted: (1) study and patient characteristics (first author, year of publication, country, study design, sample size, follow-up period for longitudinal studies, age mean/median and range, tumor entity, cancer treatment, time since diagnosis, comorbidity status), (2) results on QoL dimensions for older adults with cancer, that is QoL assessment tools, global QoL, physical QoL (PQoL), mental QoL (MQoL), social, cognitive and role functioning, and symptom scales, (3) comparison of older adults with cancer with younger counterparts and with older individuals without cancer, (4) associated factors with QoL dimensions in older adults with cancer (sociodemographic, medical, geriatric, psychological), and (5) QoL-related unmet needs and functional health aspects. No information on methodological quality of the studies was extracted, aligning with the methodological recommendations for scoping reviews from the JBI institute [21].

### Data analysis

Study characteristics and patient populations of all included studies were summarized descriptively. In studies that report results separately for older and younger adults with cancer, patient characteristics and QoL results were extracted for the older subgroup.

The different dimensions of QoL, i.e. physical QoL, mental QoL, and functioning scales (social, cognitive, role), were then summarized by synthesizing the results for older adults with cancer. A comparison of QoL between older and younger adults with cancer, as well as older cancer and older non-cancer cases was generated from the respective comparative studies. The most common associated factors were grouped into sociodemographic, medical, geriatric and psychological aspects and then summarized descriptively for each QoL dimensions. Lastly, QoL-related unmet needs and functional health aspects were categorized in order to identify overarching topics, to be presented descriptively. The data analysis and data presentation process were continuously discussed within the research team.

## Results

### Study and patient characteristics

Of the 87 articles included (see Figure 1, [22–108]), 72 employed a cross-sectional design and 15 longitudinal analysis (Table 2). The studies were conducted in 14 different countries, mostly in the United States (*n* = 43), the Netherlands (*n* = 18), Germany (*n* = 6), and France (*n* = 5). Five studies were from Asian countries. Half of the studies were published from 2016 onward, 35 studies addressed exclusively older adults with cancer, 52 studies report on age-mixed samples with either mean age ≥ 70 years or separate results for younger and older adults with cancer. Most studies were conducted among mixed (*n* = 26), breast (*n* = 21), prostate (*n* = 14), or colorectal (*n* = 13) cancer patients and sample sizes ranged from 18 to 10.1 million participants (median 477). Across all studies, the mean age was 74.2 years (range 60–107 years) for studies with exclusively older adults with cancer and 73.0 years (range 18–100 years) for age-mixed samples. Time since diagnosis ranged from newly diagnosed cancer patients to mostly 10–15 years after cancer diagnosis and most studies included all disease stages. Comorbidity burden was high with 13–94% of the patients reporting at least one comorbid condition, most commonly arthritis, cardiovascular diseases, diabetes, and hypertension. The most frequently used tools for QoL assessment were the EORTC QLQ-C30, SF-36 or SF-12. Many studies combined these general QoL assessment tools with tumor entity specific tools (e.g. EORTC QLQ-BR23). For a summary of all studies, see Supplementary Table SII.

**Table 2:** Study and pPatients’ characteristics’

Variable	*n* (%)
Study design	
Cross-sectional	72 (83)
Longitudinal	15 (17)
Country	
United States	43 (49)
Netherlands	18 (21)
Germany	6 (7)
France	5 (6)
Other	15 (17)
Sample size, median (range)	477 (18–10,076,059) participants
Age composition	
Exclusively ≥ 65 years	35 (40)
Mean (range)	74.2 (60–107) years
Age-mixed sample	52 (60)
Mean (range)	73.0 (18–100) years
Cancer entity	
Mixed	26 (30)
Breast	21 (24)
Prostate	14 (16)
Colorectal	13 (15)
Other	13 (15)
Comorbidity burden (≥ one comorbid condition)	13–94% of participants

Note: *n* (%) of included studies if not otherwise noted.

### Physical quality of life

PQoL showed a clear age pattern with declining values with advancing age [23, 26–28, 33, 38, 42, 45, 53, 61, 65, 69, 70, 73, 76–83, 85, 86, 88, 91, 100, 102–104]. A few studies argued for a curvilinear age pattern, suggesting that young-old patients may experience better PQoL than both old-old patients and younger patients (≤ 65) [38, 53]. Impairments in PQoL were often highest shortly after cancer diagnosis [27, 34, 47, 74, 88, 99] and the physical functioning of older adults with cancer thereafter often improved. Compared to older non-cancer individuals or population norms, findings indicate that older adults with cancer generally experienced either lower PQoL [27–29, 40, 43, 57, 71, 78, 87, 90, 92, 103, 105], or similar levels [23, 31, 46, 52, 56, 58, 60, 64, 82–84, 95, 108]. Notably, old-old cancer patients often reported PQoL comparable to age-matched non-cancer individuals, whereas younger patients reported poorer PQoL relative to their peers [23, 27, 40, 82, 83].

The main factors negatively impacting PQoL in older adults with cancer included the number of comorbidities [122, 24, 26, 28, 29, 31, 32, 40, 42, 47, 50, 54, 59, 61, 70, 73, 77, 80, 82, 86, 88, 90, 92, 100, 102, 104, 108], lower physical activity or an unhealthy lifestyle (e.g. smoking, obesity) [34–36, 39, 44, 63, 68, 86, 98, 100–102, 106], and increased symptom burden [29, 31, 32, 43, 73, 77, 89, 96, 107, 108]. Furthermore, lower PQoL was associated with lower education and income [22, 26, 28, 40, 73, 77, 81, 82, 86, 88, 100, 104], living alone [28, 31, 38, 40, 100], and female gender [28, 67, 88, 102, 104]. Medical and geriatric factors influencing PQoL encompassed certain cancer treatments [28, 47, 67, 73, 75, 80, 84, 94, 103, 105] (e.g. chemotherapy, ostomy), some cancer types [28, 40, 41, 65, 88, 92, 107] (e.g. lung cancer), advanced cancer stages [61, 70, 73, 77, 80, 88], impairments in activities of daily living (ADL) or reduced autonomy [79, 80, 88, 104], as well as cognitive or physical frailty [59, 78, 85, 88].

### Mental quality of life

Overall, older adults with cancer exhibited good levels of MQoL [25, 30, 47, 81]. In comparison to younger cancer patients, the vast majority of studies indicate that older patients had comparable [33, 38, 45, 69, 76, 82, 83, 85, 105] or even better [26, 30, 37, 43, 53, 65, 70, 77, 78, 81, 83, 88, 91] MQoL. Older patients appeared to adapt well mentally to a cancer diagnosis, demonstrating resilience and the capacity to overcome health and life crises [38]. Some studies showed impaired MQoL shortly after diagnosis, which then improved thereafter [26, 27, 38, 73, 74]. A study examining the temporal pattern of MQoL across different age groups demonstrated that values tended to equalize over time; older patients generally maintained a higher and stable level from the outset, while younger patients exhibited worse MQoL shortly after the cancer diagnosis, which improved over time [30]. Among older patients, there were no changes in MQoL as patients aged from young-old to old-old [23, 28, 40, 53], while some studies even indicated better values in the old-old group [81, 88].

Similarly, in comparison to cancer-free individuals or population norms, most studies showed comparable [23, 27, 52, 56–58, 64, 83, 95, 103, 105] or even better [29, 32, 60, 78, 84, 108] MQoL in cancer patients. However, a few studies reported worse MQoL in cancer patients [28, 43, 46, 57, 71, 92], which was often limited to subgroups, such as young-old patients, certain cancer types (e.g. lung or prostate cancer), or smokers.

Again, high comorbidity was one of the main factors negatively influencing MQoL [22, 26, 28, 32, 40, 42, 47, 54, 70, 77, 80, 82, 88, 92, 102, 104], even though the effect was less pronounced than on PQoL [22, 29]. In addition, MQoL in older adults with cancer tended to be strongly impacted by socioeconomic inequalities, such as income, education and occupation [22, 26, 28, 32, 40, 42, 45, 61, 73, 77, 78, 80, 88, 104], and with lower values in women [24, 28, 42, 50, 67, 78, 79, 102], patients with less social support [32, 47, 98], and patients living alone [79, 81]. Geriatric aspects such as impairments in ADL and reduced autonomy [79, 80, 88, 104], experiences of ageism or negative attitudes from healthcare professionals [77, 108], and pessimistic attitudes toward own aging [78] were associated with worse MQoL. Several medical factors reduced MQoL, including lower physical activity or an unhealthy lifestyle [34, 39, 57, 68, 102, 106], higher symptom burden [29, 32, 43, 77, 89, 108], and advanced cancer stage [28, 40, 55, 70, 73, 82, 87, 88, 92]. In addition, psychological factors tended to improve MQoL, including resilience and better coping skills [29, 47, 78], optimism or satisfaction with life [47], resourcefulness [61, 73], and communication about the disease and side effects [58, 70, 108].

### Functioning

SF in older adults with cancer revealed mixed results. Some studies showed better values with advancing age [37, 38], possibly due to less social avoidance and fewer social challenges such as childcare or work. Some studies showed comparable [76, 82, 83, 85, 105] or even worse SF in older patients [23, 27, 33, 45, 86], possibly due to social isolation and small social networks of older adults. This is also reflected in associated factors with a protective factor preventing decline in SF being physical activity and fitness [34, 39, 44, 68, 85, 86, 98, 102, 106], which may strengthen social activities and social participation. Again, comorbidity and symptom burden in older adults with cancer worsened SF [42, 54, 58, 70, 82, 86, 95, 102, 107]. Compared to non-cancer cases, older adults with cancer mostly reported worse [28, 52, 87, 103, 105] or comparable SF [23, 27, 46, 58, 64, 82–84], however the impact of cancer on younger cancer patients’ SF seemed to be more pronounced [23, 46, 95].

Role functioning (RF), including difficulties in daily activities due to emotional and physical health problems, was reported by some studies to show no age effect [83, 85, 91, 105]. However, the RF generally tended to decline with advancing age [23, 27, 33, 45, 76, 77, 79, 81, 82, 86, 103], also when comparing older to younger patients with cancer. This decline may be attributed to the increased number of comorbidities [24, 30, 42, 54, 70, 77, 82, 86, 96, 102] and reduced physical activity and fitness [34, 63, 68, 71, 85, 86, 101, 102, 106], which may result in challenges to fulfill role expectations. However, similar to PQoL, differences between cancer and non-cancer groups were often negligible [23, 27, 34, 46, 56, 58, 64, 82–84], particularly in the old-old subgroup. However, some studies reported worse RF outcomes in cancer patients compared to their cancer-free peers [28, 46, 52, 71, 87, 103, 105].

Cognitive functioning (CF) remained relatively stable [23, 25, 64, 83, 95] or showed a slight decline [30, 33, 45, 47, 69, 103] with advancing age. CF was most affected shortly after cancer diagnosis and during acute cancer treatment [46, 75, 99], and tended to improve over time. However, the direct impact of cancer and its treatment on CF was generally more pronounced in younger patients with cancer, while older patients appeared to demonstrate greater resilience [30, 83, 95, 105]. When compared to older non-cancer individuals, CF in older adults with cancer, especially in the old-old group, was largely comparable [46, 64, 83, 95].

### Unmet needs and functional health aspects

An important QoL-related unmet need for geriatric cancer patients involves effective symptom management within a coordinated healthcare approach [40, 48, 55, 58, 59, 70, 77, 82, 90, 108]. Decreasing PQoL, high rates of comorbidities and symptom burden were common in older patients; however, many patients refrained from discussing these symptoms with their doctors, often assuming they are a normal part of aging and something they must simply endure [58, 108]. In addition, older cancer patients underutilized healthcare services [82] that may help with unmet symptoms. Compounding this issue is ageism in healthcare, manifested in assumptions that certain symptoms are age-typical, communication barriers due to cognitive decline, paternalistic decision-making, and limited patient involvement in treatment choices [55, 77, 108].

Furthermore, lower social support and limited social participation represented unmet needs for older adults with cancer, directly affecting their QoL [32, 45, 47, 98, 98]. Especially impairments in physical activity, symptom burden and comorbidities severely impacted patients’ functional health, such as impairments in ADL or the risk of decline into frailty [29, 54, 59, 62, 71, 80, 88, 104]. While physical activity had well-documented benefits for PQoL, social participation played an equally vital role in supporting MQoL in older adults with cancer [98].

## Discussion

This scoping review summarized dimensions of QoL in older adults with cancer. Our findings indicate that physical burden is high in older adults with cancer, contributing to lower PQoL with advancing age. MQoL, on the contrary, tends to be stable or even improves with age, likely due to resilient coping strategies and high mental functioning. These trends align with observations in other disease populations and healthy adults [109–111]. Our results further show that CF and RF are stable or decrease with age, whereas results on SF are mixed.

In our review, PQoL among cancer patients demonstrates a clear age-related decline. Notably, younger and young-old cancer patients exhibit worse PQoL compared to their non-cancer peers, while old-old cancer patients show comparable levels to their age-matched counterparts. This suggests that the decline in PQoL among older adults with cancer might be partly attributable to the effects of normal aging rather than solely to cancer. PQoL in our results was mainly affected by factors such as comorbidity, physical activity, lifestyle choices and symptom burden. These results highlight key challenges for survivorship care and underscore the need for a comprehensive, multidisciplinary approach that addresses the unique health issues faced by geriatric patients [112, 113]. It is essential to assess patients beyond their cancer, identifying various comorbid conditions, vulnerabilities, and well-known geriatric and psychosocial risk factors that affect overall physical health.

In contrast to PQoL, MQoL does not demonstrate a clear age-related pattern in our results, with most included studies showing comparable or even better MQoL in older adults with cancer. This might be explained by a strengthened resilience with age as a combination of biological, psychological, and social factors [114, 115]. In addition, previous research has shown that older adults exhibit enhanced coping mechanisms and a greater ability to navigate adversity, which can be attributed to their accumulated life experiences and physiological adaptations [116–118]. On the other hand, due to the lack of tools to assess QoL specifically for older adults with cancer, the assessment of mental functioning in geriatric contexts may not yet be appropriate. Comparisons with older non-cancer individuals in our results suggest that cancer patients partially experience better MQoL, potentially due to personal growth stemming from their experiences with cancer and the challenges they have overcome. Socioeconomic factors, e.g. education level, income, and social support, appear to exert a greater influence on MQoL than tumor characteristics, particularly among older adults with cancer, as they have to navigate retirement, potential financial constraints or social isolation. Also previous research has demonstrated socioeconomic health disparities among cancer patients, affecting both physical and mental health [119–121]. Thus, despite the overall robust MQoL among older adults with cancer, our findings highlight the importance of addressing both psychological well-being and social health disparities in this older population.

Our findings further indicate that older adults with cancer underreport QoL-related symptoms and often do not seek effective support for their needs. This might be explained by internalized beliefs that these symptoms are normal for their age and have to be endured, which is further compounded not only by the existing ageism in oncological healthcare but also beyond the oncological field. Late- and long-term effects of cancer are potentially not attributed to the cancer disease but rather to aging. This may also be exacerbated by paternalistic patient–doctor communication, limited provision of information and the decision-making process, which could hinder effective intervention for QoL. Ageism in all medical fields and healthcare components and how it may impact QoL and related unmet needs, however, is an understudied field [122].

In addition, our findings highlight the need to improve older patients’ social support and social participation. Older patients may require additional support for managing daily life and healthcare needs, e.g. obtaining medication or travelling to medical appointments. Limited social participation, feelings of loneliness or retirement have been shown to lead to a diminished sense of purpose and deterioration in QoL [123]. This is underscored by our heterogenic picture of SF in older adults with cancer, indicating that SF might be dependent on patient characteristics, e.g. differences not only in young-olds and old-olds in social roles regarding work and care for (grand-)children but also potential cultural differences in social participation of older generations.

### Clinical implications

The findings of this study underscore the necessity for improving QoL and comprehensive survivorship care among older adults with cancer, which is increasingly relevant given our aging population. This may be achieved through prevention strategies and increased prehabilitation and rehabilitation efforts in order to preserve functional health, and ensure that older adults remain engaged in daily activities and social networks. This can be achieved through self-management digital tools [124], community-based support [125], primary care and specialized support services. In addition, geriatric assessment and individualized care is essential in this context, allowing clinicians to tailor survivorship care to unique needs. To provide holistic care, it is not only vital to support QoL through a multi-level approach including prevention of frailty, i.e. cognitive and physical decline, falls or impairments in ADL, symptom management and lifestyle interventions but also tailored support should be provided for socioeconomic vulnerabilities and functional impairment. Finally, shared decision-making emerges as a crucial component in the care of older patients, as they often do not report their physical symptom burden, which they perceive as normal for their age, feel excluded from treatment decisions or are dissatisfied with information provision. Addressing this gap through shared decision-making fosters autonomy and engagement, which can improve patient satisfaction and health outcomes.

### Strengths and limitations

The main strength of this study that enhances its validity and applicability is its high generalizability. We incorporated data from multiple healthcare settings, all tumor diagnoses, as well as studies comparing QoL of older adults with cancer to younger adults with cancer and older individuals without cancer. In addition, patients at various time points after cancer diagnosis, across different tumor stages and treatment regimen were included. Despite these strengths, several limitations must be acknowledged. Firstly, the study may be subject to publication bias, is limited to English-language articles and only included two databases. However, we identified a substantial number of studies that could be included in our review, providing a great amount of valuable data. Secondly, there is a potential for a healthy survivor bias, as individuals with poorer QoL may be less likely to participate in studies. This non-participation could skew the findings toward more favorable outcomes and results must be interpreted with regard to this potential bias. Thirdly, the reporting of cancer stage and treatment varies greatly between studies and it is therefore difficult to draw any valid conclusions on its impact on QoL. Fourthly, many studies underreport null results, which can lead to biased conclusions on differences between age groups and associated factors. Finally, contradictory results across studies indicate that subgroup analyses are necessary to adequately address specific research questions. Without these narrower analyses, it may be challenging to draw definitive conclusions that are applicable across all populations or contexts.

## Conclusion

The findings of this study show that older adults with cancer show decreasing PQoL, whereas MQoL remains stable or even increases. A comprehensive and multidisciplinary approach is essential for addressing the needs of older adults with cancer, including a thorough geriatric assessment to evaluate individual comorbidity burden, care needs, and risk factors, in addition to enhancing social support and functional health. This might help to significantly improve both the functional health and the physical and mental health for our growing aging population.

## Supplementary Material

Quality of life in older patients with cancer and related unmet needs: a scoping review

## Data Availability

The datasets generated during and/or analyzed during this study are available from the last author upon reasonable request.
