# Supplementary material for: Quality of life in older patients with cancer and related unmet needs: a scoping review
Source: Acta Oncol. 2025 Apr 15;64:42602. doi: 10.2340/1651-226X.2025.42602 (PMC12012652; doi:10.2340/1651-226X.2025.42602)
Supplement: Quality of life in older patients with cancer and related unmet needs: a scoping review [file AO-64-42602-s1.pdf]

Supplementary material has been published as submitted. It has not been copyedited, or typeset by Acta Oncologica

Table S1. Preferred Reporting Items for Systematic reviews and Meta-Analyses extension for Scoping Reviews (PRISMA-ScR) Checklist

SECTION	ITEM	PRISMA-ScR CHECKLIST ITEM	REPORTED ON PAGE #
**TITLE**			
Title	1	Identify the report as a scoping review.	1
**ABSTRACT**			
Structured summary	2	Provide a structured summary that includes (as applicable): background, objectives, eligibility criteria, sources of evidence, charting methods, results, and conclusions that relate to the review questions and objectives.	2
**INTRODUCTION**			
Rationale	3	Describe the rationale for the review in the context of what is already known. Explain why the review questions/objectives lend themselves to a scoping review approach.	3
Objectives	4	Provide an explicit statement of the questions and objectives being addressed with reference to their key elements (e.g., population or participants, concepts, and context) or other relevant key elements used to conceptualize the review questions and/or objectives.	4
**METHODS**			
Protocol and registration	5	Indicate whether a review protocol exists; state if and where it can be accessed (e.g., a Web address); and if available, provide registration information, including the registration number.	5
Eligibility criteria	6	Specify characteristics of the sources of evidence used as eligibility criteria (e.g., years considered, language, and publication status), and provide a rationale.	5
Information sources*	7	Describe all information sources in the search (e.g., databases with dates of coverage and contact with authors to identify additional sources), as well as the date the most recent search was executed.	5
Search	8	Present the full electronic search strategy for at least 1 database, including any limits used, such that it could be repeated.	5 (link to published protocol)
Selection of sources of evidence†	9	State the process for selecting sources of evidence (i.e., screening and eligibility) included in the scoping review.	5, 6
Data charting process‡	10	Describe the methods of charting data from the included sources of evidence (e.g., calibrated forms or forms that have been tested by the team before their use, and whether data charting was done independently or in duplicate) and any processes for obtaining and confirming data from investigators.	6
Data items	11	List and define all variables for which data were sought and any assumptions and simplifications made.	6
Critical appraisal of individual sources of evidence§	12	If done, provide a rationale for conducting a critical appraisal of included sources of evidence; describe the methods used and how this information was used in any data synthesis (if appropriate).	NA
Synthesis of results	13	Describe the methods of handling and summarizing the data that were charted.	7
**RESULTS**			
Selection of sources of evidence	14	Give numbers of sources of evidence screened, assessed for eligibility, and included in the review, with reasons for exclusions at each stage, ideally using a flow diagram.	5, 6
Characteristics of sources of evidence	15	For each source of evidence, present characteristics for which data were charted and provide the citations.	8
Critical appraisal within sources of evidence	16	If done, present data on critical appraisal of included sources of evidence (see item 12).	NA
Results of individual sources of evidence	17	For each included source of evidence, present the relevant data that were charted that relate to the review questions and objectives.	Supplement Table S2
Synthesis of results	18	Summarize and/or present the charting results as they relate to the review questions and objectives.	8-11
**DISCUSSION**			

SECTION	ITEM	PRISMA-ScR CHECKLIST ITEM	REPORTED ON PAGE #
Summary of evidence	19	Summarize the main results (including an overview of concepts, themes, and types of evidence available), link to the review questions and objectives, and consider the relevance to key groups.	12, 13
Limitations	20	Discuss the limitations of the scoping review process.	14
Conclusions	21	Provide a general interpretation of the results with respect to the review questions and objectives, as well as potential implications and/or next steps.	14, 15
**FUNDING**			
Funding	22	Describe sources of funding for the included sources of evidence, as well as sources of funding for the scoping review. Describe the role of the funders of the scoping review.	25

JBI = Joanna Briggs Institute; PRISMA-ScR = Preferred Reporting Items for Systematic reviews and Meta-Analyses extension for Scoping Reviews.

* Where *sources of evidence* (see second footnote) are compiled from, such as bibliographic databases, social media platforms, and Web sites.

† A more inclusive/heterogeneous term used to account for the different types of evidence or data sources (e.g., quantitative and/or qualitative research, expert opinion, and policy documents) that may be eligible in a scoping review as opposed to only studies. This is not to be confused with *information sources* (see first footnote).

‡ The frameworks by Arksey and O'Malley (6) and Levac and colleagues (7) and the JBI guidance (4, 5) refer to the process of data extraction in a scoping review as data charting.

§ The process of systematically examining research evidence to assess its validity, results, and relevance before using it to inform a decision. This term is used for items 12 and 19 instead of "risk of bias" (which is more applicable to systematic reviews of interventions) to include and acknowledge the various sources of evidence that may be used in a scoping review (e.g., quantitative and/or qualitative research, expert opinion, and policy document).

From: Tricco AC, Lillie E, Zarin W, O'Brien KK, Colquhoun H, Levac D, et al. PRISMA Extension for Scoping Reviews (PRISMAScR): Checklist and Explanation. Ann Intern Med. 2018;169:467–473. doi: 10.7326/M18-0850.

Table S2. Summary of included studies (n = 87)

#	Authors	Country	Study design	QoL assessment tool	Sample	Cancer sites	QoL results
1	Aarts et al., 2010	Netherlands	Population-based cross-sectional study	SF-36	*N*=584, age-mixed sample, mean age = 75.1 (low SES), 74.5 (medium SES), 74.0 (high SES), range 50-80+ years	Prostate cancer	Socioeconomic inequalities exist especially in mental QoL and borderline effects in physical QoL; Comorbidity affects both, physical and mental QoL
2	Adam et al., 2020	Germany	Population-based cross-sectional study	EORTC QLQ-C30 / -PR25	*N*=1,975, age-mixed sample, mean=73.0 years, range 50-89 years, disease free	Prostate cancer	QoL is comparable in older cancer patients (≥75) compared to cancer-free controls; physical and role functioning scales decline with age irrespective of cancer; emotional functioning gets better with older age; cancer patients report more diarrhea, constipation and insomnia, but less pain
3	Allareddy et al., 2006	United States	Population-based cross-sectional study	FACT-G / -BL	*N*=259, age-mixed sample, mean=73.3 years, long-term survivors	Bladder cancer	Cancer treatment had no effect on QoL in bladder cancer, apart from sexual dysfunction; older age and comorbidity lowers QoL
4	Arraras et al., 2018	Spain	Longitudinal study (follow-up 3 years)	EORTC QLQ-C30 / -BR23	*N*=173, exclusively elderly cancer patients (≥65), mean=72.1 years, treated with radiotherapy	Breast cancer	QoL is high across time in early-stage breast cancer, indicating good adaptation to disease (only moderate limitations in sexual functioning and enjoyment); no clinically relevant differences in QoL between treatment groups
5	Babcock et al., 2020	United States	cross-sectional study	SF-12	*N*=10.1 million, around 48% ≥65 years	Mixed cancer	Polypharmacy influences physical but not mental QoL; physical QoL gets worse with age, whereas mental QoL gets better with age
6	Baker et al., 2009	United States	Longitudinal study (follow-up 2 years)	SF-36	*N*=7,838, exclusively elderly cancer patients (≥65), comparison to *N*=9,012 cancer-free controls	Mixed cancer	Cancer patients have worse physical QoL than cancer-free controls and comparable mental QoL; deteriorations across time in the same amount, but newly diagnosed cancer patients deteriorate faster over time
7	Baker et al., 2003	United States	cross-sectional study	SF-36	*N*=22,747, exclusively elderly cancer patients (≥65), comparison to *N*=22,747 cancer-free controls	Mixed cancer	Older cancer patients have worse values than cancer-free controls on all QoL domains; physical and mental QoL is influenced by several sociodemographic and medical factors
8	Beck et al., 2009	United States	Longitudinal mixed methods	SF-12	*N*=52, exclusively elderly cancer patients	Mixed cancer	Older cancer patients have worse physical and better mental QoL compared to population norms over time;

#	Authors	Country	Study design	QoL assessment tool	Sample	Cancer sites	QoL results
			study (follow-up 3 months)		(≥65), mean age = 71.5 years, range 65-81 years, comparison to population norms		high symptom burden over time (fatigue, pain, sleep disturbances, etc.)
9	Bantema-Joppe et al., 2015	Netherlands	Longitudinal study (follow-up 5 years)	EORTC QLQ-C30 / -BR23	*N*=1,420, different age groups, range 28-85 years, 16% ≥70	Breast cancer	Younger cancer patients show worse role, emotional and cognitive functioning, as well as fatigue and pain shortly after radiotherapy compared with older cancer patients, but improve over time; worse sexual functioning in older patients across time compared to younger patients
10	Bellury et al., 2013	United States	cross-sectional study	SF-36	*N*=184, exclusively elder cancer patients (≥70), mean age = 76.6, range 70-95 years	Breast cancer	Physical functionality is strongly influenced by symptom bother, comorbidity and marital status
11	Bellury et al., 2012	United States	cross-sectional study	SF-36	*N*=759, exclusively elder cancer patients (≥70), mean age = 77.6, range 70-98 years	Breast cancer	Physical QoL mainly impacted by symptom bother, comorbidity, social support and marital status; mental QoL mainly impacted by education, age, social support and comorbidity
12	Beutel et al., 2015	Germany	population-based cross-sectional study	EORTC QLQ-C30	*N*=689, separate results for age groups, 36.7% ≥70 years	Malignant melanoma	Decline of general QoL an all functioning scales (except emotional functioning) with older age; symptoms increased with older age
13	Blair et al., 2016	United States	cross-sectional study	SF-36	*N*=1,776, exclusively older cancer patients (≥70), mean age = 78.6 years, range 73-88, comparison to *N*=12,599 cancer free controls	Mixed cancer	Physically active cancer patients are not at risk for poorer QoL. Physically inactive cancer patients (especially with shorter time since diagnosis) have worse general health, vitality and physical function
14	Boisen et al., 2016	New Zealand	cross-sectional study	WHOQOL-BREF / -OLD	*N*=137, age-mixed sample, mean age 72.5-75.9 years	Prostate cancer	Physical QoL is better in physically active patients; psychological, social and environmental QoL is not impacted by physical activity
15	Breedveld-Peters et al., 2018	Netherlands	cross-sectional study	EORTC QLQ-C30	*N*=155, age-mixed sample, mean age = 70 years	Colorectal cancer	Adherence to lifestyle recommendations is associated with better physical QoL and less fatigue; in women and obese patients it additionally was associated with better general QoL and less disability
16	Carreira et al., 2021	United Kingdom	Population-based cross-sectional study	QLACS	*N*=356, separate results for age groups, range 34-81 years, 34.6% ≥70	Breast cancer	Older cancer patients have fewer negative feelings, cognitive problems, sexual dysfunction, social avoidance, financial problems, family- and recurrence-related distress, and thus better global QoL compared to younger cancer patients ≤60 years

#	Authors	Country	Study design	QoL assessment tool	Sample	Cancer sites	QoL results
17	Cimprich et al., 2002	United States	cross-sectional study	QOL-CS	*N*=105, separate results for age groups, range 34-89 years, 27% ≥66 at diagnosis	Breast cancer	Patients with older age at diagnosis show worse physical QoL than younger patients, however with a curvilinear pattern; older patients report better social well-being and no difference on psychological and spiritual QoL; older patients feel less useful in life and have a higher uncertainty about the future
18	Clark et al., 2008	United States	cross-sectional study	LASA QOL	*N*=272, age-mixed sample, mean age = 71 years, range 27-95 years	Lung cancer	Physically active patients have better QoL on all dimensions (overall, physical, mental, emotional, social, spiritual)
19	Clauser et al., 2008	United States	cross-sectional study	SF-36	*N*=21,504, exclusively older cancer patients (≥65), mean age = 70 years	Mixed cancer	Older cancer patients have worse physical QoL than cancer-free controls, but the effect was attenuated for old-old patients (≥85); mental QoL was lower in cancer patients, however, effect was smaller than on physical QoL
20	Clough-Gorr et al., 2010	United States	Longitudinal study, (follow-up 7 years)	SF-36	*N*=400, exclusively older cancer patients (≥65)	Breast cancer	Cancer patients with symptoms of persisting lymphedema report worse physical function and emotional health than patients without lymphedema over a period of 7 years
21	Clouth et al., 2021	Netherlands	population-based longitudinal study (follow-up 2 years)	EORTC QLQ-C30	*N*=1,489, age-mixed sample, mean age = 70.5 years	Colon cancer	Five QoL classes were identified that are timely stable: majority has excellent (37%) or good QoL (with insomnia 32%, with physical limitations 9%), moderate QoL with limitations in function scales and fatigue (14%), poor QoL with severe limitations in functioning and symptoms (8%)
22	Couderc et al., 2023	France	cross-sectional study	SF-12	*N*=371, separate results for age groups, range 18-82 years, 21% ≥70	Lung cancer	Factors that influence QoL are age-dependent; 43% of older patients (≥70) have impaired physical and 19% impaired mental QoL compared to age-matched population norms; older patients are less likely to have impaired QoL than younger patients
23	Dialla et al., 2015	France	cross-sectional study	SF-12, EORTC QLQ-C30 / -BR23	*N*=396, separate results for age groups, range 27-96 years, 34% ≥65	Breast cancer	Older cancer patients have worse functioning scales and physical QoL, however comparable mental QoL with younger cancer patients; determinants of QoL are age-dependent (for older cancer patients comorbidity and socioeconomic deprivation)
24	Doege et al., 2019	Germany	Population-based cross-sectional study	EORTC QLQ-C30	*N*=2,647, separate results for age groups, range 30-89 years, 37% ≥70, comparison to	Breast cancer	Compared to cancer-free controls, QoL domains in cancer patients are impaired only in some age groups; patients ≥80 mostly have comparable QoL with cancer-free controls

#	Authors	Country	Study design	QoL assessment tool	Sample	Cancer sites	QoL results
					N=1,005 cancer free controls		
25	D'Silva et al., 2018	Australia	cross-sectional study	FACT-L, TOI	*N*=127, age-mixed sample, mean age = 71 years	Lung cancer	Sedentary time and physical activity related to physical QoL, global QoL and fatigue
26	Durá-Ferrandis et al., 2017	United States	Longitudinal study (follow-up median 4.5 years, up to 7 years)	EORTC QLQ-C30	*N*=1,280, exclusively older cancer patients (≥65), mean age = 72.7 years, range 65-91 years	Breast cancer	Most women maintain their QoL over time; deterioration in emotional functioning (6.9%), cognitive functioning (7.6%), and physical functioning (31.8%); coping strategies, social support influenced the QoL trajectories
27	Faul et al., 2014	United States	Longitudinal study, (follow-up 2 years)	EORTC QLQ-C30	*N*=328, exclusively older cancer patients (≥65), mean age = 72.8 years, range 65-88 years	Breast cancer	Survivorship care plans are not associated with physical, emotional and role functioning
28	Galalae et al., 2004	Germany	cross-sectional study	EORTC QLQ-C30, PSM-G	*N*=145, age-mixed sample, mean age = 73 years, range 47-86 years	Prostate cancer	Adjuvant hormonal therapy impacts global QoL, social functioning and prostate specific symptom scales (sexuality, constipation, heat flushes, financial problems)
29	Gemmill et al., 2010	United States	cross-sectional study	mCOH-QOL-O	*N*=307, age-mixed sample, mean age = 74 years, range 38-95 years	Mixed cancer with urinary diversion	Continence in patients with urinary diversions influences QoL issues such as skin problems, family distress and financial worries
30	Gopalakrishna et al., 2018	United States	cross-sectional study	FACT-BL	*N*=459, age-mixed sample, mean age = 74 years	Bladder cancer	Diet quality was significantly associated with QOL in univariate analysis but loses statistical significance after adjustment for potential confounders.
31	Hammerlid et al., 2001	Sweden	Population-based cross-sectional study	SF-36, EORTC QLQ-C30 / -H&N35	*N*=135, separate results for age groups, range 18-83 years, 43% ≥65	Head and neck cancer	Older adults with cancer (≥65) have worse clinically important differences in most QoL domains (mainly role-physical and social functioning) compared to age-matched controls, whereas younger patients have similar values compared to age-matched controls
32	Harden et al., 2008	United States	cross-sectional study	SF-12, EPIC	*N*=69, separate results for age groups, range 50-80 years	Prostate cancer	Young-old patients (65-74) experience better physical QoL than middle-aged (50-65) and old-old (75-84), as well as better mental QoL than middle-aged
33	Harrison et al., 2017	United States	Population-based cross-sectional study	SF-36, VR-12	*N*=924, exclusively older cancer patients (≥65)	Breast cancer	Cancer patients with heart failure are at risk for deficits in QoL across all domains; the magnitude varied by cancer stage: physical QoL impaired in all stages, mental QoL mainly impaired in early-stage breast cancer with heart failure

#	Authors	Country	Study design	QoL assessment tool	Sample	Cancer sites	QoL results
34	Harrison et al., 2011	United Kingdom	cross-sectional study	EQ-5D	*N*=659, age-mixed sample, mean age = 71.6 years, range 42-92 years	Mixed cancer	Long-term survivors have comparable QoL when compared to population norms
35	Hart et al., 2018	Canada	Population-based cross-sectional study	FACT-G	*N*=296, age-mixed sample, mean age = 73.2 years, comparison to *N*=255 cancer free controls	Colorectal cancer	Long term survivors have comparable QoL than cancer-free controls, and even better global and social QoL
36	Hays et al., 2008	United States	Population-based cross-sectional study	SF-36	*N*=123,567, exclusively older cancer patients (≥65), mean age = 75.3 years, range 65-107	Mixed cancer	Cancer patients have worse physical and mental QoL compared to non-cancer cases, which is further influenced by smoking status (smokers have worse QoL)
37	Heidrich et al., 2006	United States	cross-sectional study	SF-36	*N*=18, exclusively older cancer patients (≥65), mean age, 74.2 years, comparison to *N*=24 cancer free controls	Breast cancer	No difference in QoL between cancer and non-cancer patients; patients attributed most of their symptoms to aging
38	Ho et al., 2023	Hong Kong	cross-sectional study	EORTC QLQ-C30	*N*=293, exclusively older cancer patients (≥65), mean age = 70.1 years	Mixed cancer	Survivors with cognitive frailty have worse global health status, physical and social functioning, and fatigue
39	Hoogland et al., 2021	United States	cross-sectional study (mixed methods)	SF-12	*N*=56, exclusively older cancer patients (≥65), mean age = 72.5 years, range 62-87 years	Mixed cancer	Older cancer patients have positive psychological changes with higher mental QoL compared to population norms, but comparable physical QoL
40	Huang et al., 2018	Taiwan	cross-sectional study	SF-36, EORTC QLQ-PR25,	*N*=200, age-mixed sample, mean age = 73.2 years	Prostate cancer	Patients with greater resourcefulness show better physical, mental and prostate-specific QoL
41	Jackson et al., 2019	United Kingdom	Longitudinal study (follow-up 6 years pre to 2 years post diagnosis)	CASP-19	*N*=477, age-mixed sample, mean age = 71.4 years, comparison to *N*=5,451 cancer free controls	Mixed cancer	Global QoL decreases around 0-2 years pre cancer diagnosis up until 2 years after cancer diagnosis compared to cancer-free controls
42	Karvinen et al., 2007	Canada	cross-sectional study	FACT-BL	*N*=525, age-mixed sample, mean age = 70.2 years	Bladder cancer	Physical exercise is positively associated with QoL in a dose-response relationship for global, physical, functional, and bladder cancer specific QoL
43	Kerleau et al., 2016	France	Population-based cross-sectional study	EORTC QLQ-C30, EPIC	*N*=287, exclusively older cancer patients (≥60), mean age = 75.8 years,	Prostate cancer	Cancer patients report similar QoL as cancer-free controls 10 years after treatment, but higher urinary, bowel and sexual adverse effects, especially those

#	Authors	Country	Study design	QoL assessment tool	Sample	Cancer sites	QoL results
					range 61-91 years comparison to *N*=287 cancer-free controls		with treatment of radical prostatectomy and radiotherapy
44	Kornblith et al., 2007	United States	longitudinal study (follow-up 1 year)	SF-12	*N*=252, separate results for age groups, 52% ≥65	Breast and endometrial cancer	Older cancer patients have worse physical but better mental QoL compared to younger cancer patients
45	Krahn et al., 2013	Canada	Population-based cross-sectional study	FACT-P	*N*=585, age-mixed sample, mean age = 72.6 years, range 43-98 years	Prostate cancer	Comorbidity, bowel, sexual and urinary function, but also age are strongest predictors for QoL years into survivorship
46	Krouse et al., 2009	United States	cross-sectional study	SF-36, mCOH-QOL-O	*N*=491, age-mixed sample, mean age = 72.4 (ostomy), 71,1 (no ostomy) years	Rectal cancer	Ostomy influences QoL domains, however, varying effects between men and women
47	Krouse et al., 2017	United States	cross-sectional study	SF-12, COH-QOL-CRC	*N*=557, age-mixed sample, mean age = 72.6 years	Rectal cancer	Physical activity may improve QoL, particularly psychological well-being
48	Kurian et al., 2018	Netherlands	cross-sectional study	EORTC QLQ-C30 / -PR25	*N*=617, separate results for age groups, 33% ≥71 years	Prostate cancer	Older cancer patients show worse physical functioning and lower sexual activity compared to younger patients
49	Lamers et al., 2016	Netherlands	cross-sectional study	EORTC QLQ-C30 / -PR25	*N*=697, age-mixed sample, mean age = 71.3 years	Prostate cancer	Patients being dissatisfied with information provision about the disease report lower scores on all QoL dimensions
50	Lee et al., 2016	Korea	cross-sectional study	EQ-5D	*N*=1,776, exclusively older cancer patients (≥65), mean age = 72.5, age range 65-94, comparison to *N*=1,766 cancer-free controls	Mixed cancer	Older cancer patients have more problems with pain and discomfort, usual activities, mobility, and have worse values on QoL-related anxiety and depression
51	Lemij et al., 2023	Netherlands	longitudinal study (follow-up 5 years)	EORTC QLQ-C30, EORTC QLQ-BR23	*N*=239, exclusively older cancer patients (≥70), mean age = 74 years	Breast cancer	Better QoL is associated with physical activity and preservation of ability to perform activities of daily living
52	Lin et al., 2017	Taiwan	cross-sectional study	EORTC QLQ-PR25, SF-36	*N*=133, age-mixed sample, mean=74,7 years	Prostate cancer	Resourcefulness along with further sociodemographic and medical factors impact physical, mental and prostate-specific QoL
53	Litwin et al., 1998	United States	Longitudinal study (follow-up 2 years)	SF-36, UCLA Prostate Cancer Index	*N*=68, age-mixed sample, mean=71.2/75.4 years	Prostate cancer	All QoL domains improve over time after cancer diagnosis and treatment

#	Authors	Country	Study design	QoL assessment tool	Sample	Cancer sites	QoL results
54	Mak et al., 2016	United States	cross-sectional study	EQ-5D, EORTC QLQ-C30 /-BLM30, EPIC	*N*=173, age-mixed sample, mean=73 years / 76 years	Bladder cancer	Patients treated with trimodality therapy compared to radical cystectomy have better global QoL, functioning scales, bowel and sexual QoL
55	Mamguem Kamga et al., 2021	France	cross-sectional study	SF-12	*N*=145, separate results for age groups, 56% (endometrial), 38% (ovarian) ≥70 years	Mixed cancer	Older endometrial and ovarian cancer patients have worse physical and partly role functioning compared to younger patients but comparable mental, social and sexual functioning;
56	Mandelblatt et al., 2003	United States	cross-sectional study	SF-12	*N*=1,812, exclusively older cancer patients (≥67), mean age = 72.8 years	Breast cancer	Cancer treatment does not impact QoL in older breast cancer patients; several sociodemographic and medical factors impact QoL
57	Martin et al., 2019	United States	cross-sectional study	SF-36	*N*=171, age-mixed sample, mean age = 80.1, age range 50-99, comparison to *N*=683 cancer-free controls	Mixed cancer	Optimistic attitude towards own aging increases physical and mental QoL; older cancer patients have worse physical, but better mental QoL compared to cancer-free controls
58	Matsuda et al., 2003	France	Population-based cross-sectional study	FACT-G, FACT-BI	*N*=95, age-mixed sample, mean age = 72 years, range 33-99 years	Bladder cancer	Cancer patients with poor autonomy and disadvantaged family situations have impairments in QoL domains
59	Mogal et al., 2017	United States	Population-based longitudinal study	VR-12	*N*=373, exclusively older cancer patients (≥65), mean age = 74.6 years	Breast cancer	Inability to perform activities of daily living is strongest predictor for physical and mental QoL when controlled for confounders
60	Mols et al.,2008	Netherlands	Population-based cross-sectional study	SF-36, UCLA Prostate Cancer Index (EPIC)	*N*=590, exclusively older cancer patients (≥60), mean age = 74.9 (with diabetes), 74.1 (without diabetes) years, range 60-85 years	Prostate cancer	Cancer patients with diabetes have worse general health and vitality than cancer patients without diabetes, and worse general health compared to a matched normative population
61	Mols et al., 2007	Netherlands	Population-based cross-sectional study	SF-36	*N*=1,112, separate results for age groups, 58% ≥70, comparison to *N*=1,742 cancer-free controls	Mixed cancer	Lower physical functioning in older cancer patients compared to younger cancer patients probably age-related because there is no difference between older cancer and age-matched cancer-free controls; older cancer patients have comparable QoL as age-matched cancer-free controls
62	Mols et al., 2018	Netherlands	Population-based cross-sectional study	EORTC QLQ-C30, THYCA-QOL	*N*=293, separate results for age groups, 13% ≥65, range 18-84 years,	Thyroid cancer	Older cancer patients have worse physical functioning and more thyroid-specific symptoms than younger cancer patients; however compared to an age-

#	Authors	Country	Study design	QoL assessment tool	Sample	Cancer sites	QoL results
					comparison to *N*=586 cancer-free controls		matched cancer-free population there were no differences in QoL domains
63	Mols et al., 2006	Netherlands	Population-based cross-sectional study	SF-36, QoL-CS	*N*=780, exclusively older cancer patients (≥60), range 60-85 years, compared to age-matched norm population	Prostate cancer	Patients with radical prostatectomy report highest physical QoL five to ten years after diagnosis compared to radio and hormone therapy; compared to an age-matched norm population, cancer patients have worse general health but better mental health scores
64	Morishita et al., 2022	Japan	cross-sectional study	SF-36	*N*=102, separate results for age groups, 48% ≥65 years	Breast cancer	Older breast cancer patients have worse physical functioning, and comparable other QoL domains as middle-aged patients; association between muscle strength / balance and QoL domains is mainly observed in older breast cancer patients
65	Mosher et al., 2009	United States	cross-sectional study	SF-36	*N*=753, exclusively older cancer patients (≥65), mean age = 73 years, range 65-87 years	Mixed cancer	Older age, less education, more comorbidities are associated with reduced physical functioning and role limitations; exercise, dietary habits and body weight are associated with physical and role functioning
66	Moss et al., 2021	United States	Population-based cross-sectional study	SF-36, VR-12	*N*=95,627, exclusively older cancer patients (≥65), mean age = 74,7 years, comparison to *N*=176,013 cancer-free controls	Mixed cancer	Cancer patients have worse QoL domains compared to controls; the reduction in QoL differed by rurality and cancer types
67	Pandya et al., 2016	United States	population-based longitudinal study	VR-12	*N*=17,958, exclusively older cancer patients (≥65)	Mixed cancer	Older cancer patients have worse physical and mental QoL, as well as greater decline with a history of falls; several sociodemographic and medical factors are associated with QoL
68	Roiland et al., 2011	United States	Secondary analysis of RCT baseline	SF-36	*N*=192, exclusively older cancer patients (≥65), mean age = 70 years, range 65-97 years	Breast cancer	Higher physical symptom is associated with worse physical and mental QoL
69	Siembida et al., 2021	United States	Population-based cross-sectional study	PROMIS	*N*=2,019, exclusively older cancer patients (≥65), mean age = 71.8 years	Mixed cancer	High rate of multi-morbidity in older cancer patients; specific comorbid conditions lead to different QoL impairments
70	Sio et al., 2014	United States	cross-sectional study	EORTC QLQ-C30, EORTC QLQ-BR23	*N*=402, separate results for age groups, range 29-97 years, 30% ≥65 years	Breast cancer	Older cancer patients report poorer general health, and physical functioning compared to younger patients, but have a better body image and less concerns about recurrence and dying

#	Authors	Country	Study design	QoL assessment tool	Sample	Cancer sites	QoL results
71	Smith et al., 2008	United States	Population-based cross-sectional study	SF-36	*N*=14,897, exclusively older cancer patients (≥65), mean age = 75.3 years, comparison to *N*=111,788 cancer-free controls	Mixed cancer	Cancer patients have worse physical QoL and partly worse mental QoL compared to non-cancer cases; comorbidities have a negative impact on QoL
72	Sun et al., 2016	United States	cross-sectional study, mixed methods	mCOH-QOL-O	*N*=575, age-mixed sample, mean age = 74 years, range 36-100 years	Rectal cancer	Cancer patients with an ostomy have lower overall QoL and have more sexual QoL problems (e.g. personal relationships, being intimate, appearance)
73	Swanick et al., 2018	United States	Population-based cross-sectional study	EQ-5D-3L, BREAST-Q	*N*=489, exclusively older cancer patients, range 67-87 years at diagnosis	Breast cancer	Patients with less irradiation and less surgery report better long-term physical, psychosocial and sexual QoL
74	Thong et al., 2019	Germany	Population-based cross-sectional study	EORTC QLQ-C30	*N*=1,262, separate results for age groups, mean age = 70 years, comparison to *N*=1,689 cancer-free controls	Colorectal cancer	Long-term survivors report mostly comparable QoL domains compared to cancer-free controls and impairments are mainly in younger patients; however increased symptoms such as diarrhea and constipation remain present in cancer patients
75	Thong et al., 2019	Germany	Population-based cross-sectional study	EORTC QLQ-CR29	*N*=1,176, separate results for age groups, 49% 60-69, 18% ≥70 years	Colorectal cancer	Older cancer patients compared to younger patients have higher functioning (future perspective, body image); results on symptoms are mixed (less buttock pain, more hair loss, more urinary frequency, less sexual interest)
76	Thong et al., 2018	Netherlands	cross-sectional study	EORTC QLQ-C30	*N*=1,183, age-mixed sample, mean age = 71 years	Mixed cancer	Patients with fatigue score worse on all QoL functioning scales, as well as global QoL
77	Thraen-Borowski et al., 2013	United States	cross-sectional study	SF-36	*N*=832, exclusively older cancer patients (≥65), mean age = 81.5 years	Colorectal cancer	Physical activity (even non-exercise, light-intensity) in older cancer patients is related to physical QoL, while social participation is related to mental QoL
78	Ubels et al., 2015	Netherlands	longitudinal study (follow-up 5 years)	EORTC QLQ-C30, EORTC QLQ-LC13	*N*=39, age-mixed sample, median age = 77 years, range 55-87 years	Lung cancer	Level of global health was maintained over 5 years; functioning scores fluctuate over time
79	Utley et al., 2022	United States	cross-sectional study	RAND-36	*N*=173, exclusively older cancer patients (≥65), mean age = 73.5 years	Mixed cancer	Physical function, BMI and social support are inter-correlated with bodily pain
80	van Roekel et al., 2016	Netherlands	cross-sectional study	EORTC QLQ-C30	*N*=145, age-mixed sample, median age = 70 years	Colorectal cancer	Substituting sedentary time with standing or physical activity increases physical QoL, and is associated with better fatigue

#	Authors	Country	Study design	QoL assessment tool	Sample	Cancer sites	QoL results
81	van Veen et al., 2019	Netherlands	cross-sectional study	EORTC QLQ-C30	*N*=1,096, age-mixed sample, median age = 70.8 years, 76% ≥65 years	Colorectal cancer	Higher adherence to diet and physical activity guidelines is associated with better physical, role, cognitive social functioning, global health status, and fatigue
82	van Walree et al., 2019	Netherlands	Population-based cross-sectional study	EORTC QLQ-C30	*N*=191, separate results for age groups, 31% ≥ 70 years, comparison to *N*=264 cancer-free controls	Ovarian cancer	Older cancer patients have worse global health and all functioning scales (except emotional) and symptoms than older cancer-free individuals; older cancer survivors have worse global health, physical, role functioning and symptoms compared to younger cancer patients; older survivors with chemotherapy have better physical QoL, pain and fatigue, whereas younger survivors with chemotherapy have worse values on these scales
83	Vang et al., 2023	United States	cross-sectional study	SF-36, VR-12	*N*=10,488, exclusively older cancer patients (≥65), mean age = 76.5 years	Mixed cancer	Race has an impact on mental but not physical QoL with Asian American having worse mental QoL values compared to non-Hispanic White patients
84	Verweij et al., 2018	Netherlands	Population-based cross-sectional study	EORTC QLQ-C30, EORTC QLQ-CR38	*N*=2,299, separate results for age groups, 66% (≥65), comparison to *N*=239 cancer-free controls	Colorectal cancer	No difference in QoL domains between age groups; impact of ostomy on QoL domains is less pronounced in elderly patients compared to younger patients (<65); older cancer patients show worse social and physical functioning, and symptoms than normative population
85	Vissers et al., 2017	Netherlands	Population-based cross-sectional study	EORTC QLQ-C30	*N*=1,111, age-mixed sample, mean age = 70.7 years	Colorectal cancer	BMI and waist circumference are associated with global health, functioning and fatigue
86	White et al., 2014	United States	Population-based cross-sectional study	SF-36, VR-12	*N*=9,282, exclusively older cancer patients (≥65)	Mixed cancer	The co-occurrence of major depression and urinary incontinence has a large negative impact on role emotional and social functioning, as well as other QoL domains; lung cancer patients are particularly at risk
87	Yeom et al., 2009	United States	cross-sectional study	SF-12	*N*=61, exclusively older cancer patients (≥65), mean age = 71 years, range 65-86 years	Breast cancer	Older breast cancer patients who perceived more negative attitudes from their healthcare professionals and reported more communication difficulties report lower levels of mental QoL
